# Psychosocial Predictors of Suicidal Thoughts and Behaviors in Mexican-Origin Youths: An 8-Year Prospective Cohort Study

**DOI:** 10.1177/21677026221102924

**Published:** 2022-12-01

**Authors:** Lauren C. Gonzalves, Emilio Ferrer, Richard W. Robins, Amanda E. Guyer, Paul D. Hastings

**Affiliations:** 1 Department of Psychology, University of California, Davis; 2 Center for Mind and Brain, University of California, Davis; 3 Department of Human Ecology, University of California, Davis

**Keywords:** suicidality, psychosocial predictors, Mexican-origin youths, adolescents

## Abstract

Suicide is the second leading cause of death for youths in the United States. More Latino adolescents report suicidal thoughts and/or behaviors (STBs) than youths of most other ethnic communities. Yet few studies have examined multiple psychosocial predictors of STBs in Latino youths using multiyear longitudinal designs. In this study, we evaluated the progression of STBs in 674 Mexican-origin youths (50% female) from fifth grade (10 years old) to 12th grade (17 years old) and identified psychosocial predictors of changes in STBs across this period. Latent growth curve models revealed that being female and later-generation status were associated with increasing prevalence in STBs across adolescence. Family conflict and peer conflict predicted increased STBs, whereas greater familism predicted less STBs. Thus, interpersonal relationships and cultural values contribute to the development of STBs in Mexican-origin youths and may be key levers for decreasing suicidality in this understudied but rapidly growing portion of the U.S. adolescent population.

Suicide is the second leading cause of death among all youths ages 10 to 19 in the United States (Centers for Disease Control and Prevention [CDC], 2017), and rates have nearly doubled in the last decade (Hoffmann et al., 2020). This is especially evident for Latino youths in general (Khan et al., 2018; Miron et al., 2019; Twenge et al., 2019) and particularly Latina youths, who have higher rates of suicidal thoughts and/or behaviors (STBs) than youths of most other ethnic and racial communities (Kann et al., 2014; Price & Khubchandani, 2017; Villarreal-Otálora et al., 2019; Zayas et al., 2005). Despite increasing rates of STBs in Latino adolescents, factors that may be driving this trend remain uncertain because most prior research has used cross-sectional or short-term longitudinal studies and single-predictor analyses. In California, Latino youths constitute the majority of children ages 0 to 17 years (State of California, Department of Finance, 2019), and the large majority identify Mexico as their family’s country of origin (i.e., “Mexican origin”; Public Policy Institute of California, 2019). Therefore, identifying factors that contribute to the development of STBs across adolescence in Mexican-origin youths is essential to inform the design of appropriate interventions with this rapidly growing and high-risk population (Ford-Paz et al., 2015; Standley, 2020).

To help mitigate the rising rate of adolescent suicidality in the United States, developmental scientists focused on suicide have called for research to (a) expand how STBs are defined and conceptualized, (b) focus on individual and malleable mechanisms, (c) investigate developmental trajectories of risk factors related to STBs, and (d) make greater efforts to account for diverse populations (Cha et al., 2018). Many risk factors for suicidality among Latino youths are similar to those identified for adolescents of other ethnicities (e.g., identifying as female, interpersonal conflict; Eaton et al., 2006; Kuhlberg et al., 2010), but culture-specific predictors have also been identified, such as ethnic discrimination (Cheref et al., 2019; Hwang & Goto, 2008), generation status (Peña et al., 2008; Pottie et al., 2015), and less endorsement of traditional cultural values (Kuhlberg et al., 2010; Peña et al., 2011). In this study, we examined ethnic discrimination, generation status, and familism as factors that may be particularly relevant for adolescent suicidality in Latino communities and family conflict, peer conflict, and sex as factors shown to be associated with adolescent suicidality across communities. Although some past research has studied various subsets of these variables simultaneously (Kuhlberg et al., 2010; Peña et al., 2011), none to our knowledge have modeled each of these risk and protective factors in a single model. Furthermore, we used a longitudinal design spanning ages 10 to 17 years to examine whether there were specific ages at which these factors contributed to risk for STBs across adolescence in Mexican-origin youths.

## Predictors of STBs for Latino Adolescents

Adolescence is recognized as a period of rapid social and biological changes that cumulatively contribute both to opportunity for growth (NASEM, 2019) and greater risk for psychopathology (Miller & Prinstein, 2019). To understand youth suicidality in Latino communities specifically, researchers must consider the family sociocultural environment in which these developmental changes unfold (Zayas et al., 2005). Indeed, a culturally informed developmental perspective in the examination of STBs in Latino youths necessarily approaches its examination of other potentially contributing factors within the context of cultural factors (Duarté-Vélez & Bernal, 2010).

Zayas and colleagues (2005) proposed a conceptual model of Latina STBs that highlights the significance of examining distal environmental factors, including both culturally specific (e.g., values) and nonspecific factors (e.g., family functioning), as establishing the context of vulnerability within which proximal antecedent factors (e.g., interpersonal crises) trigger STBs. In this framework, our examination of ethnic discrimination, generation status, and familism reflect such culturally specific distal factors, whereas sex, family conflict, and peer conflict constitute distal factors affecting many communities. These factors may foster either risk for or resilience against STBs, but importantly, in this sample and community, both kinds of distal factors will have been experienced by Mexican-origin youths who simultaneously stand outside the dominant culture yet must exist in that society, which is often hostile to people who are perceived and categorized as immigrants or minority members. Hence, consistent with intersectionality frameworks (Crenshaw, 2017), past studies of STB in majority culture youths (i.e., European American Whites) cannot be assumed to inform the unique and distinct ways in which culture, sex, and interpersonal relationships forge contexts of risk and resilience that are experienced by Mexican-origin or other Latino youths (Zayas et al., 2005).

### Ethnic discrimination

Ethnic discrimination encompasses direct and systemic treatment of people on the basis of their perceived nonmajority social classification that disadvantages them and defines them as inferior to or different than members of the majority group (Blank et al., 2004; Jones, 2000). Nearly 40% of Latino respondents living in the United States report experiencing direct ethnic or racial discrimination (e.g., being told to return to their home country, being criticized for speaking Spanish; Lopez et al., 2018). The deleterious impact of perceived discrimination on mental health is well established (Araújo & Borrell, 2006; Arora & Wheeler, 2018; Stein et al., 2019; Trent et al., 2019; Vines et al., 2017). Latino youths who experience more ethnic discrimination report more STBs (Cervantes et al., 2014; Cheref et al., 2019; Gomez et al., 2011; Hwang & Goto, 2008), yet documented associations have tended to be of small to medium magnitude.

Given that adolescents spend more waking hours per day in peer and school contexts compared with other venues (Vernon, 2005) and the importance of peer-group relationships for adolescent identity development and well-being (La Greca & Harrison, 2005; Ragelienė, 2016), experiencing ethnic discrimination from peers and teachers may be particularly pernicious for Latino youths (Tormala et al., 2015). A retrospective study of Latino middle school and high school students found that ethnic-based bullying was related to increased suicidal ideation for depressed youths (Cardoso et al., 2018). Hence, in this prospective investigation, we focused on Mexican-origin adolescents’ experiences of ethnic discrimination in school settings.

### Generation status and acculturation

Many Mexican-origin adolescents in the United States must navigate the dominant culture into which the current or prior generations of their family have immigrated, and generation status is a powerful predictor of psychopathology (Peña et al., 2008; Pottie et al., 2015). The “immigrant paradox” holds that those U.S. residents with foreign nativity—immigrants—are relatively protected against psychopathology than are subsequent U.S.-born generations (Alegría et al., 2008). Numerous studies have documented an association between later-generation status and poorer mental-health outcomes in both Latino youths (García Coll & Marks, 2012) and adults (Alegría et al., 2008; Grant et al., 2004). Peña et al. (2008) found that compared with first-generation Latino youths, second-generation Latino youths were more than twice as likely to attempt suicide, and third-generation youths were more than 3 times as likely. Likewise, in a national sample, the percentage of U.S.-born Latinas at risk for suicide was a quarter greater than the percentage of first-generation (immigrant) adolescent Latinas who reported STBs (Substance Abuse and Mental Health Services Administration [SAMHSA], 2003).

Generation status is not synonymous with acculturation, yet internalization of majority-culture values increases across generations, and there are associations between acculturation and STBs in youths and young adults of some communities (Castle et al., 2011; Ng, 1996; Ortin et al., 2018). This may act through interpersonal processes, because later-generation youths often experience greater child–parent cultural dissonance, and resulting family conflict, because the youths may prefer certain U.S. majority-culture attitudes or practices (Ying & Han, 2007). Conversely, first-generation youths often identify more with traditional family values (Canino & Roberts, 2001), which may reduce family conflict and buffer against the stresses associated with acculturation and assimilation (Cervantes et al., 2014). As Mexican-origin families continue to immigrate to the United States and stay for generations, examining whether the immigrant paradox is present for Mexican-origin youths will be important to inform both future research and current interventions.

### Familism

Compared with White individuals of Western European descent, people of Mexican-origin and other Latino communities typically live in larger family networks, are more family-oriented, and have greater expectations of exchange and reciprocity among family members (Keefe, 1984; Mindel, 1980). A multidimensional construct, “familism” encompasses both the strong identification or bond with the nuclear and extended family and feelings of familial loyalty and solidarity (Marín & Marín, 1991). A robust sense of familism, in which members greatly depend on one another for several needs, may buffer Latino adolescents from developing STBs. Latino adolescents who report greater familism also report better mental health (Kapke et al., 2017; Stein et al., 2015), and suicide attempts are less prevalent among Latina youths who reported tight-knit families with strong familism compared with youths who do not (Kuhlberg et al., 2010; Peña et al., 2011). Few studies have examined direct relations between familism on STBs, and to date, studies have been cross-sectional such that it remains unclear whether familism protects against STBs over time.

### Family conflict

Family conflict is related to STBs across cultures (Kuhlberg et al., 2010; Lau et al., 2002; E. Miller et al., 2012), and this form of interpersonal strain may create a particularly stressful environment for Latino adolescents. In many Latino cultures, the family unit is central to identity (Zayas et al., 2005), and fissures in the family can destabilize developing youths’ sense of self-worth, security, and predictability. Latino adults who reported negative family interactions and less family cohesion while growing up were more likely to report also experiencing suicidality before the age of 18 (Fortuna et al., 2007). Likewise, in a study of Latino youths, greater adolescent–parent conflict was associated with suicide attempts, although this relation was mediated by self-esteem and internalizing behaviors (Kuhlberg et al., 2010).

There is abundant research documenting the association between family conflict and adolescent STBs across all communities (Asarnow & Carlson, 1988; Brent et al., 1994; Carballo et al., 2020; Fergusson & Lynskey, 1995). In a sample of Asian American children and adolescents, participants who reported high parent–child conflict were nearly 30 times more likely to engage in suicidal ideation and/or self-harm compared with their peers who reported lower levels (Lau et al., 2002). Prinstein and colleagues (2000) reported a concurrent link between adolescent reports of overall family dysfunction and increased suicidal ideation in a clinically high-risk sample of 12- to 17-year-olds, and effect sizes neared medium magnitude. Likewise, DeVille et al. (2020) found that in a sample of approximately 12,000 9- and 10-year-old children, participants living in high-conflict families were more likely to report suicidal ideation and/or to have attempted suicide compared with peers living in lower-conflict families. This association held after controlling for family history of depression and suicidality, financial adversity, and parent-reported child internalizing and externalizing problems, suggesting that dysfunction in the parent–child relationship may be specifically relevant for identifying risk for STBs. However, with a paucity of longitudinal research examining associations between family conflict and STBs in Latino populations, it is unclear whether family conflict is a prospective risk factor contributing to the development of suicidality in Latino adolescents. Given the centrality of the familial unit in Latino communities, including the Mexican-origin population, experiences of family conflict warrant longitudinal examination.

### Peer conflict

The quality of peer relationships is also an important predictor of adolescent suicidality (Grudnikoff et al., 2015; Prinstein et al., 2000). Interpersonal stressors, such as being bullied or excluded by peers, are commonly reported by youths who also engage in STBs (Cheek et al., 2020; Juvonen & Graham, 2014; King & Merchant, 2008; Winsper et al., 2012). More conflict with peers has been associated with a greater likelihood of endorsing STBs in Latinas, specifically (Romero et al., 2013), and both Latino and Latina adolescents who reported peer victimization at school evinced higher levels of suicidality compared with their peers who did not report being victimized (Robinson et al., 2021; Villarreal-Otálora et al., 2020). In a cross-cultural meta-analysis, Van Geel and colleagues (2014) found a robust, concurrent association between peer victimization and adolescent STBs across dozens of studies; yet the authors also noted the lack of prospective research. Thus, greater conflict with peers is a risk factor for STBs in Latino youths, yet it remains an open question whether and at what age experiencing peer conflict constitutes a specific risk factor for the onset of STBs in Mexican-origin youths.

### Biological sex

STBs are more prevalent among adolescent girls of all ethnic groups compared with adolescent boys (Kann et al., 2014, 2018; Rich et al., 1992), especially Latinas (Kann et al., 2014; Price & Khubchandani, 2017; Rasmussen et al., 1997; Villarreal-Otálora et al., 2019; Zayas et al., 2005). Across extant studies, rates of completed suicides for adolescent girls are lower than those for adolescent boys in several industrialized countries, but suicidal ideation and attempts follow an opposite trend (Fergusson et al., 2000; Grunbaum et al., 2004). That is, although girls may be less likely to die by suicide, they are more likely to endorse suicidal ideation and engage in nonlethal suicidal behaviors (Lawson et al., 2022; Orri et al., 2020; Rich et al., 1992). This well-documented sex gap may widen when examining ethnic-minority populations. Across epidemiological studies, adolescent Latinas have steadily evinced elevated rates of STBs, higher than their male counterparts and adolescents of most other ethnicities (Querdasi & Bacio, 2021). In the United States, an estimated 15.6% of Latina adolescents have attempted suicide one or more times (CDC, 2013); until recently, this problem garnered little attention in the medical or mental-health fields (Nock et al., 2013).

## Development of STBs in Adolescence

Most longitudinal research on adolescent STBs has used predominantly Euro-American samples, neglecting the ethnic and racial diversity of youths in the United States. Studies of the developmental course of STBs have found that suicidality increases, on average, from early to late adolescence, with moderate to high rank-order stability, indicating that prior suicidality predicts future suicidality (Carbone et al., 2019; Garrison et al., 1991; Orri et al., 2020; Steinhausen et al., 2006). Suicidal ideation peaks around age 15 (Rueter & Kwon, 2005), near the transition into high school. Likewise, epidemiological data suggest that the highest rate of STBs occurs between 12 and 17 years of age (Nock et al., 2013). Together, these studies suggest that adolescence represents a uniquely vulnerable window for STBs, and recent national data (Ivey-Stephenson et al., 2020; Ruch et al., 2019) indicate that suicidality is on the rise in this age group with no signs of abating, but less is known about the developmental course of suicidality in Latino youths.

In our prospective study of Mexican-origin youths, we conducted annual assessments of STBs beginning at age 10. Our first article on STBs in Mexican-origin youths (Lawson et al., 2022) documented increases over early adolescence that peaked between 14 and 15 years, mirroring the developmental trajectory of adolescent STBs in other ethnic groups (Nock et al., 2013). Focused on temperamental predictors of STBs, that study revealed that effortful control was associated with decreased probability of STBs by Mexican-origin youths, whereas negative emotionality (e.g., aggression, frustration, depressed mood) was associated with increased likelihood of STBs. In the current investigation, we examined the degree to which six salient risk and protective factors predicted the onset and developmental course of STBs in Mexican-origin youths.

## The Present Study

In the current work, we focused on psychosocial predictors specific to the lives of Mexican-origin youths across their adolescent years, from ages 10 to 17. Most prior research on the predictors of STBs in Latino youths have been cross-sectional examinations of concurrent associations (i.e., Cardoso et al., 2018; Kann et al., 2018; Piña-Watson et al., 2014) or brief longitudinal studies of 1- to 2-year durations (i.e., Roche et al., 2020). Additional longitudinal repeated measures studies across adolescence are required to inform interventions with Mexican-origin youths because it is unknown whether multiple psychosocial factors differentially predict STBs at particular points in adolescence. Therefore, using an 8-year prospective design, in the current investigation, we aimed to (a) measure the onset and growth of STBs in Mexican-origin youths across the adolescent years and (b) examine which factors contribute to presence versus absence of STBs at different points of adolescent development.

We hypothesized that, in our sample of Mexican-origin adolescents, (a) female youths would evince a faster rate of increasing STBs over adolescence compared with males; (b) later-generation youths would evince a faster rate of increasing STBs over adolescence; (c) greater family conflict, peer conflict, and ethnic discrimination would be concurrently and prospectively associated with reporting STBs; and (d) greater familism would act as a protective factor, decreasing the prevalence of reported STBs concurrently and prospectively. These risk and protective factors were assessed repeatedly across adolescence, but because of the scarcity of prior developmentally informed research, we did not pose a priori hypotheses about whether they would be associated with STBs more versus less strongly at different ages.

## Method

### Participants

In this study, we used data from the California Families Project (CFP), a longitudinal study of 674 Mexican-origin youths (50% female) in northern California focused on predicting the emergence and development of substance use. Youths were drawn at random from the 2006–2007 and 2007–2008 school rosters of fifth-grade classrooms in one large (population > 450,000) city and one small (population < 60,000) city that served adjacent rural areas. The majority of the students enrolled in these two school districts were Latino (i.e., 53% Latino, 20% White, 11% Asian, 10% African American, and 6% other racial or ethnic backgrounds). Youth and parent participants self-identified as being of Mexican heritage, and youths were 29% first generation (born in Mexico, immigrated to United States), 62% second generation (born in United States to parents who immigrated to United States), and 9% third generation (grandparents were born in Mexico and immigrated to United States). Most families were two-parent households (82%); gross annual household income ranged from less than $5,000 to more than $95,000, measured in $5,000 increments (*M* = $32,500). Annual family income was converted into income-to-needs (ITN) ratios on the basis of family size. Parents provided informed consent for their child’s participation, and youths provided assent. This study was approved by the university’s Institutional Review Board.

Youths were assessed annually from fifth grade (age: *M* = 10.86 years, *SD* = 0.50) through 12th grade (age: *M* = 17.73 years, *SD* = 0.52). Retention was high, based on youths who participated in 3 or more years of data collection (94.8%; *N* = 639). The sample sizes for each assessment were as follows: Grade 5, *N* = 674; Grade 6, *N* = 569 (84.4%); Grade 7, *N* = 578 (85.8%); Grade 8, *N* = 591 (87.7%); Grade 9, *N* = 605 (89.8%); Grade 10, *N* = 590 (87.5%); Grade 11, *N* = 600 (89.0%); and Grade 12, *N* = 600 (89.0%). No youths were lost because of suicide or other causes of death during this period.

### Procedures and measures

Trained research staff interviewed participants in their homes in either Spanish or English, according to participant preference. Interviewers were all bilingual, and most were of Mexican heritage. To ensure discretion and given the sensitive nature of the questions about STBs, youths responded to the measures assessing STBs without the help of the interviewers (i.e., youths reported their responses directly on a computer that was turned away from interviewers), apart from the administration of a clinical interview that was implemented as a part of the larger CFP study, discussed below.

#### STBs

To measure participants’ STBs, two different instruments were used: the National Institute of Mental Health Diagnostic Interview Schedule for Children (DISC-IV; Shaffer et al., 2000) and a brief suicide questionnaire adapted from the Youth Risk Behavior Survey (Brener et al., 2013). The DISC-IV is a fully structured diagnostic interview designed for use by nonclinicians to measure common psychiatric symptoms for diagnoses in children (Shaffer et al., 2000). In the annual assessments from fifth grade through 12th grade, youths responded (0 = no, 1 = yes) to two DISC-IV items assessing STBs (i.e. “Was there a time when you thought seriously about killing yourself?” and “During the last year, have you tried to kill yourself?”). The suicide questionnaire was administered annually from sixth grade through 12th grade and included three self-report items: “Have you thought about committing suicide?” “Have you made a plan for committing suicide?” and “Have you attempted suicide?” (1 = *never*, 2 = *once*, 3 = *twice*, 4 = *3 or more times*).

After visits were completed and interviews were examined by research staff, parents were contacted if youths reported having any imminent thoughts of self-harm and/or suicide. Although study questions did not specifically probe for imminent risk, if youths spontaneously reported active suicidality, research staff contacted caregivers and made appropriate referrals.

A dichotomous score for STBs was derived from the two DISC-IV items in fifth grade plus the three additional items in all subsequent years. Participants’ affirmative responses to one or more of the items were scored as 1 (yes) for STBs at that grade. Participants responding never or no to all items were scored as 0 (no) for STBs at that grade. The intraclass coefficient for the five items used to generate the final score for STBs ranged from α = .66 to α = .78 (*M* = .73)

#### Psychosocial predictors

Each psychosocial predictor variable was measured by youths’ self-report at Grades 5, 7, 9, and 11, allowing for concurrent and prospective predictions of how each factor may uniquely exacerbate or mitigate participants’ STBs across adolescence.

##### Family conflict and peer conflict

Conflict with family and conflict with peers were assessed using two subscales from the Multicultural Events Scale for Adolescents (MESA; Gonzales et al., 1995). To measure family conflict, participants answered yes (1) or no (0) to nine items assessing recent (i.e., past 3 months) negative interactions with caregivers (i.e., “You had a serious disagreement or fight with a parent”; mean coefficient αs across grades ranged from .56 to .69, *M* = .64). To measure peer conflict, participants answered yes (1) or no (0) to 14 items assessing recent negative life events involving peers (i.e., “Other kids said mean or bad things to you”; αs = .60–.73, *M* = .67). For each scale, responses were averaged at each grade such that higher scores indicated greater conflict (possible range = 0.0–1.0). Given that the MESA is a checklist of individual events, test-retest reliability may be a better indicator of internal validity, and test-retest correlations for a 2-week period demonstrated strong reliability for the complete measure (*rs* = .69–.81; Gonzales et al., 1995).

##### Ethnic discrimination

Ethnic discrimination was measured using six items from the Adolescent Perceptions of Discrimination scale (Johnston & Delgado, 2004), which assessed discrimination specifically targeted at Mexicans and Mexican Americans in the school setting (e.g., “You have heard kids at school making jokes or saying bad things about Mexicans or Mexican-Americans”). Participants rated each item from 1 (*not at all true*) to 4 (*very true*). Responses were averaged at each grade (αs = .54–.71, *M* = .63); higher scores indicate more perceived ethnic discrimination (possible range = 1.0–4.0).

##### Familism

Familism was measured using 16 items from the Mexican-American Cultural Values Scale (MACVS; Knight et al., 2010), which assesses felt support, obligation, and need to defer to the nuclear family (e.g., “How much do you agree that parents should teach their children that the family always comes first?”). Participants rated each item from 1 (*not at all*) to 4 (*very much*). Responses were averaged at each grade (αs = .82–.88, *M* = .86); higher scores indicate greater familism (possible range = 1.0–4.0).

### Data analytic plan

To examine changes in STBs over time and predict STBs levels at each grade, we used latent growth curve (LGC) modeling (Bollen & Curran, 2006; McArdle, 2009). LGC models estimate within-persons trajectories of growth and between-persons differences in these trajectories. We examined whether these trajectories were a function of time-invariant and time-variant theoretically derived predictors (Raudenbush et al., 2001). Observed STBs across grades (5–12) were treated as a dichotomous dependent variable (1 = yes, 0 = no). Each measure of STBs was then used to create a latent variable at each grade representing a continuous latent response underlying the observed yes or no responses (Masyn et al., 2013). Changes in the latent variables across grades were examined with the LGC. All models were implemented in Mplus (Muthén & Muthén, 2018) using the weighted least squares mean (i.e., WLSM) adjusted estimator.

In the first set of LGC models, we tested no-growth, linear-growth, and latent-basis models to assess the trajectory that provided the best fit for STBs across grades. Model fit was evaluated using four indices: (a) the χ^2^ goodness-of-fit test, (b) the comparative fit index (CFI; Bentler, 1990), (c) the Tucker-Lewis index (TLI; Tucker & Lewis, 1973), and (d) the root mean square error of approximation (RMSEA; Steiger, 1990). In these analyses, we did not include ITN ratio as a covariate because preliminary analyses using zero-order correlations and structural equation models found that ITN ratios were not significantly associated with adolescent STBs.

In the second set of LGC models, the goal was to examine the role of psychosocial factors. We included these variables first individually and then simultaneously. These variables were added as time-varying covariates to predict the latent factor representing STBs concurrently (Grades 5, 7, 9, 11) and prospectively (Grades 6, 8, 10, 12) after accounting for the stability over time of STBs. Therefore, each psychosocial predictor was examined for its concurrent association with STBs and for its prediction of increased or decreased STBs in the subsequent year.

First, we examined four LGC models, including each psychosocial predictor separately to examine the individual contributions to changes in STBs over time, while adjusting for sex and generation status as time-invariant covariates. Second, the four psychosocial predictors then were included in one LGC model to examine unique and additive contributions to STBs while we adjusted for sex and generation status as time-invariant covariates.

Missing data ranged from 5% to 16% in each year. To account for this, we used full information maximum likelihood (i.e., FIML). FIML is highly flexible in dealing with partially random missing data (Graham et al., 2007) and one of the most efficient and unbiased estimations techniques for missing data currently available (Enders & Bandalos, 2001). Little’s (1988) missing completely at random test indicated that data were not missing completely at random, χ^2^(638) = 756.21, *p* < .01. Attrition analysis for multiple comparisons revealed no differences between participants who were present versus absent in Grade 12 across the 16 measures of focal variables. Missingness was not associated with demographic characteristics, such as socioeconomic status, generation status, or sex, indicating that the data conformed to missing at random (i.e., Enders, 2010) standards.

## Results

### Descriptive statistics

**Fig. 1.**
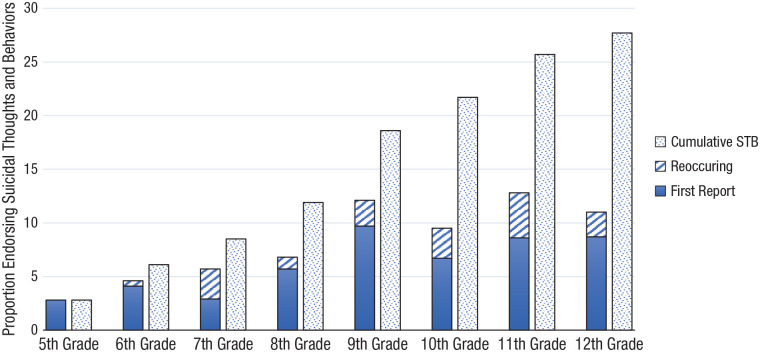
Proportion of participants endorsing a history of suicidal thoughts and/or behaviors (STBs) beginning at Grade 5. STBs was measured annually, and history of STBs was not collected before fifth grade. Reoccurring and first report represent the total proportion of participants who endorsed STBs at that grade. Cumulative STBs represents participants who endorsed at the current or prior grade. Proportions represent the valid percentage, based on the number of participants at each grade. See Table 1 for sample sizes at each grade.

Descriptive statistics for the prevalence of STBs are reported in Table 1 and for psychosocial predictors in Table 2. STBs increased from Grades 5 (age: *M* = 10.9 years) to 9 (age: *M* = 14.8 years) and then was maintained at comparable prevalence through to Grade 12 (age: *M* = 17.7 years); more girls than boys reported STBs from Grade 6 onward. By Grade 12, 27.7% of the total sample (38% of girls; 17.5% of boys) had endorsed STBs at least once, and 13.7% (20.2% of girls; 7.5% of boys) had endorsed at two or more grades. Considering generation status, we found that 16.8% of first-generation, 22.4% of second-generation, and 20% of third-generation youths had reported STBs by Grade 12. Figure 1 illustrates the proportion of participants who disclosed initial and/or reoccurring STBs at each grade and the cumulative proportion of participants who endorsed having a history of STBs across the eight grades.

**Table 1.** Summary Information on Participants’ Reporting of Suicidal Thoughts and/or Behaviors at Each Grade

Grade	Mean age (years)	Total *N*	Endorsed suicidality *N* (%)	Females endorsing *N* (%)	Males endorsing *N* (%)	Prior suicidality *N* (%)	Subsequent suicidality *N* (%)
5	10.9	642	18 (2.8)	8 (2.5)	10 (3.1)	—	7 (38.9)
6	11.8	565	26 (4.6)	16 (5.7)	10 (3.5)	3 (11.5)	19 (73.1)
7	12.8	577	33 (5.7)	25 (8.6)	8 (2.8)	17 (51.5)	26 (78.8)
8	13.8	591	40 (6.8)	32 (10.7)	8 (2.7)	17 (42.5)	25 (62.5)
9	14.8	605	73 (12.1)	55 (18.0)	18 (6.0)	28 (38.4)	52 (71.2)
10	15.8	590	56 (9.5)	42 (14.0)	14 (4.8)	35 (62.5)	35 (62.5)
11	16.8	600	77 (12.8)	57 (18.9)	20 (6.7)	50 (64.9)	38 (49.4)
12	17.7	600	66 (11.0)	46 (15.1)	20 (6.8)	52 (78.8)	—

Note: Values reported include total number and proportion reporting STBs, number and proportion of females and males reporting STBs, and number and proportion of youths reporting STBs who had reported STBs in the prior years and who reported STBs in the subsequent years. STBs = suicidal thoughts and/or behaviors.

**Table 2.** Regression Coefficients of Four Latent Growth Curve Models Predicting Suicidal Thoughts and/or Behaviors From Fifth Grade to 12th Grade

		STBs								
	Grade		5th	6th	7th	8th	9th	10th	11th	12th
Predictor		*M* (*SD*)	*B* (*SE*)							
Family conflict										
	5th	0.08 (0.13)	1.81* (0.77)	0.19 (1.27)	—	—	—	—	—	—
	7th	0.07 (0.12)	—	—	3.07*** (0.44)	0.58 (0.49)	—	—	—	—
	9th	0.10 (0.15)	—	—	—	—	2.07*** (0.34)	0.65 (0.50)	—	—
	11th	0.10 (0.15)	—	—	—	—	—	—	1.53*** (0.40)	1.26* (0.49)
Peer conflict										
	5th	0.15 (0.16)	1.52* (0.66)	−0.25 (2.04)	—	—	—	—	—	—
	7th	0.48 (0.34)	—	—	0.02 (0.17)	−0.02 (0.14)	—	—	—	—
	9th	0.13 (0.15)	—	—	—	—	2.12*** (0.33)	0.43 (0.49)	—	—
	11th	0.12 (0.14)	—	—	—	—	—	—	0.83 (0.51)	0.48 (0.56)
Discrimination										
	5th	1.40 (0.40)	0.26 (0.12)	−0.08 (0.30)	—	—	—	—	—	—
	7th	1.25 (0.31)	—	—	0.20 (0.15)	0.05 (0.12)	—	—	—	—
	9th	1.29 (0.33)	—	—	—	—	0.41** (0.12)	0.12 (0.35)	—	—
	11th	1.30 (0.31)	—	—	—	—	—	—	0.29* (0.12)	0.22^†^ (0.12)
Familism										
	5th	3.63 (0.31)	0.13 (0.28)	−0.19 (0.17)	—	—	—	—	—	—
	7th	3.60 (0.34)	—	—	−0.45*** (0.09)	−0.60*** (0.13)	—	—	—	—
	9th	3.52 (0.36)	—	—	—	—	−0.78*** (0.16)	−0.81*** (0.15)	—	—
	11th	3.44 (0.39)	—	—	—	—	—	—	−0.77*** (0.16)	−0.76*** (0.16)

Note: Each model was predicted individually by family conflict, peer conflict, ethnic discrimination, and familism measured at fifth, seventh, ninth, and 11th grades. The regression coefficients presented in this table are unstandardized and represent the unit change expected in STBs given a 1-unit change in the key predictor of each model, concurrently (i.e., fifth to fifth) and 1 year subsequently (i.e., fifth to sixth). Means and standard deviations reported are unstandardized values. Sex and generation status were included in each model, and slopes were specified to show growth of STBs from Grades 5 to 9 and STBs stability from Grades 10 to 12. Model fit was excellent and had the following range of values: χ^2^(65) = 80.68–138.01, .0001 < *p* < .09, comparative fit index =. 097–1.0, Tucker-Lewis index = 0.96–1.0, root mean square error of approximation = .01–.04. Significant effects are in bold. STBS = suicidal thoughts and/or behaviors.

† *p* < .10. *p < .05. **p < .01. ***p < .001.

Concurrent correlations between the time-varying predictors were computed for each year they were collected. Peer and family conflict showed the strongest correlations across each year they were collected (*r*s = .16–.55, all *p*s < .001). Experiences of ethnic discrimination were significantly and positively correlated with peer and family conflict (*r*s = .08–.41, all *p*s < .05). The correlation between familism and family conflict ranged from –.23 to –.04 and was significant at Grades 7, 9, and 11 (all *p*s < .01). Overall, correlations between predictors were small to moderate in magnitude.

### Changes in STBs across time

#### LGC with no covariates

**Fig. 2.**
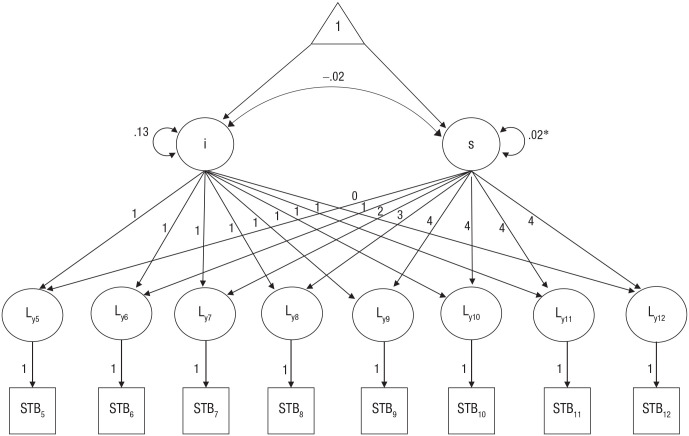
Results of an eight-wave nonlinear unconditional latent growth curve model of Mexican-origin youths’ suicidal thoughts and/or behaviors (STBs) from Grade 5 to Grade 12. Significance levels are denoted as *p < .05. The slope and intercept variances and covariance reported represent unstandardized results.

An unconditional nonlinear growth curve estimating latent STBs over time produced better model fit than the no-growth or linear-growth models, χ^2^(24) = 34.46, *p* = .08, CFI = .99, TLI = .99, RMSEA = .03 (see Fig. 2). This model yielded a linear increase in STBs from fifth grade to ninth grade, followed by stability of the prevalence of STBs across the remaining grades. The positive estimate of the slope mean (*M* = .09, *SE* = .02, *p* < .0001) together with the slope loadings (λ = 0, 1, 2, 3, 4, 4, 4, 4) indicated that individual STBs significantly increased over time until Grade 9 and then stabilized. Given that the intercept was fixed to zero, as an arbitrary initial STBs status, these changes can be interpreted as departing from zero. The variance around the mean slope was statistically significant (*p* < .05), indicating there was notable variability across participants in the development of STBs from Grades 5 to 12.

#### LGC with sex, generation status, and time-variant predictors

Psychosocial factors at Grades 5, 7, 9, and 11 were first entered independently into four separate single-predictor models (see Table 2) and then jointly into one multipredictor model (see Table 3) to examine concurrent and prospective unique and additive contributions to STBs, respectively. Here, the predictors were related to the latent factor representing STBs. In addition, autoregressive paths for STBs were also included to control for the year-to-year stability of STBs, and sex and generation status were retained in all models. All time-varying and time-invariant predictors were allowed to covary in each model.

**Table 3.** Regression Coefficients of the Latent Growth Curve Model Predicting Suicidal Thoughts and/or Behaviors From Fifth Grade to 12th Grade Predicted Jointly by Family Conflict, Peer Conflict, Ethnic Discrimination, and Familism

Suicidal thoughts and behaviors									
Grade		5th	6th	7th	8th	9th	10th	11th	12th
	Predictor	*B* (*SE*)							
5th									
	Family conflict	1.80* (0.60)	−2.21 (1.99)	—	—	—	—	—	—
	Peer conflict	0.56 (0.70)	1.41 (1.24)	—	—	—	—	—	—
	Discrimination	−0.07 (0.20)	0.53 (0.38)	—	—	—	—	—	—
	Familism	0.13 (0.29)	−0.36 (0.32)	—	—	—	—	—	—
7th									
	Family conflict	—	—	2.84*** (0.44)	0.89 (0.73)	—	—	—	—
	Peer conflict	—	—	−0.18 (0.22)	0.15 (0.20)	—	—	—	—
	Discrimination	—	—	0.35 (0.24)	−0.14 (0.23)	—	—	—	—
	Familism	—	—	−0.38*** (0.11)	−0.32** (0.11)	—	—	—	—
9th									
	Family conflict	—	—	—	—	1.35** (0.44)	0.21 (0.50)	—	—
	Peer conflict	—	—	—	—	1.05* (0.51)	1.21* (0.55)	—	—
	Discrimination	—	—	—	—	0.03 (0.18)	0.004 (0.19)	—	—
	Familism	—	—	—	—	−0.46*** (0.12)	−0.49*** (0.13)	—	—
11th									
	Family conflict	—	—	—	—	—	—	1.37** (0.44)	1.39** (0.47)
	Peer conflict	—	—	—	—	—	—	0.33 (0.63)	0.46 (0.62)
	Discrimination	—	—	—	—	—	—	−0.11 (0.22)	−0.23 (0.22)
	Familism	—	—	—	—	—	—	−0.39** (0.13)	−0.36** (0.12)

Note: This model was predicted jointly by family conflict, peer conflict, ethnic discrimination, and familism measured at fifth, seventh, ninth, and 11th grades. The regression coefficients presented in this table are unstandardized and represent the unit change expected in suicidal thoughts and behaviors given a 1-unit change in a given predictor while we adjusted for the other predictors, concurrently and 1 year subsequently. Sex and generation status were included in the model. Model fit was excellent: χ^2^(149) = 229.50, *p* < .0001, comparative fit index = .99, Tucker-Lewis index = .97, root mean square error of approximation = .03. Significant effects are in bold.

* *p* < .05. **p < .01. ***p < .001.

As presented in Table 2, when examined individually, each psychosocial predictor was concurrently associated with STBs in at least two of four grades, and family conflict and familism were prospectively predictive of STBs in one or more subsequent grades. The number and magnitude of associations increased across grades, as more adolescents reported STBs. Family conflict and familism demonstrated the greatest numbers of significant paths predicting both concurrent and subsequent STBs. The directions of all associations were in accord with predictions: More family and peer conflict and more ethnic discrimination predicted higher levels of STBs, whereas greater familism predicted lower levels of STBs. Across the models with individual predictors, generation status was a significant predictor of the slope in every model, and sex was a significant predictor of the slope in two of the four models; later-generation and female youths evinced steeper slopes for STBs. Each single-predictor model had excellent model fit (for specific indices, see Table 2).

As presented in Table 3, when psychosocial predictors were examined simultaneously in one LGC model, family conflict and peer conflict significantly and positively predicted STBs both concurrently and prospectively, whereas familism significantly and negatively predicted STBs concurrently and prospectively. Family conflict and familism predicted STBs across most grades; peer conflict in ninth grade was specifically linked to greater STBs in ninth grade and 10th grade. In contrast, ethnic discrimination was not significantly related to STBs at any age after accounting for the other predictors. These effects were incremental and additive to the significant and positive effects for sex on the slope (β = 0.07, *SE* = 0.03, *p* < .05), generation status on the slope (β = 0.06, *SE* = 0.02, *p* < .01), and year-to-year stability of STBs from Grades 6 through 11 (βs = 0.33–0.54, *SE*s = 0.09–0.14, all *p*s < .05–.0001). Female and later-generation participants had steeper trajectories of increasing STBs over time compared with male and earlier-generation participants. This model with all predictors entered simultaneously had excellent model fit (for specific indices, see Table 3).

To facilitate the interpretation of the path coefficients in Table 3, median splits were performed on the significant predictors to describe rates of STBs for youths reporting relatively higher versus lower values of the psychosocial measures. Note that these descriptive statistics are not direct translations of the path coefficients from the model but are provided to help convey the magnitudes of the effects identified. In concurrent associations, youths who reported lower familism had at least twice the prevalence of STBs as youths who reported greater familism (Grade 7: 8.1% vs. 3.6%; Grade 9: 16.2% vs. 7.8%; Grade 11: 18% vs. 8%); the opposite pattern was evident for less versus more family conflict (Grade 5: 1.1% vs. 5.4%; Grade 7: 1.6% vs. 13.8%; Grade 9: 6.6% vs. 20.4%; Grade 11: 6.2% vs. 22.4%) and peer conflict (Grade 9: 6.1% vs. 19.1%). These effects were mirrored in the prospective associations for familism (Grade 7 familism to Grade 8 STBs: 7.2% vs. 5.2%; Grade 9 to 10: 12.9% vs. 5.7%; Grade 11 to 12: 12.1% vs. 8%), family conflict (Grade 11 to 12: 4.1% vs. 21.6%), and peer conflict (Grade 9 to 10: 4.1% vs. 15.6%).

## Discussion

The emergence of STBs during adolescence for Mexican-origin youths was driven by biological sex and generation status, experiences of contentious family and peer relationships, and disconnection from the traditional Latino value of familism. More family conflict and less familism were predictive of subsequent STBs across adolescence, whereas peer conflict appeared to be particularly pernicious in the midadolescence period. Whereas ethnic-discrimination experiences at school predicted adolescent STBs when considered individually, when examined in the context of the other family- and peer-relationship measures, it was not independently and additively associated with suicidality.

Recognizing the cultural significance of relational processes for risk of STBs is critical for public- and mental-health professionals working with Mexican-origin and other Latino youths and their families, many of whom may interface with community mental-health providers. Despite being at an elevated risk for STBs (Kann et al., 2018; Villarreal-Otálora et al., 2019; Zayas et al., 2005), Latino youths are underrepresented in studies of the predictors of STBs, making these novel findings even more critical for efforts to address youths’ mental-health crisis. By identifying the antecedent risk and protective factors that predict subsequent STBs in Mexican-origin adolescents, this study builds on prior documentation of the increasing prevalence of suicidal ideation, plans, and attempts from early adolescence into emerging adulthood (Lawson et al., 2022) and reveals potentially actionable targets for sorely needed STB prevention and intervention efforts (Alegría et al., 2002).

There was both increasing STBs across adolescence, with the emergence of new cases annually, and marked stability in youths reporting STBs from year to year. Cumulatively, STBs had been reported by almost 30% of Mexican-origin youths by 12th grade. The prevalence of STBs increased more quickly in girls than boys, and more than twice as many females as males reported STBs at least once over adolescence. Likewise, in accord with the immigrant-paradox model (Alegría et al., 2008; Peña et al., 2008), STBs increased more quickly and were markedly more prevalent for youths of later-generation status compared with first-generation youths. All first-generation youths included in this study had immigrated to the United States by age 10, and thus, we might have observed even stronger effects for generation status had our sample included participants who had immigrated after their 10th birthday, given that some scholars have theorized that children who immigrate at a very young age may closer resemble second-generation youths (Perlmann, 2005).

Although the developmental course of suicidality appeared to mirror that of other ethnic and racial adolescent populations, other studies using community samples found lower lifetime prevalence rates of STBs (Orri et al., 2020; Rueter & Kwon, 2005) compared with the population present in this sample. Such an increase in suicidality across development for Mexican-origin youths, especially for girls and U.S.-born adolescents, calls for an expansion of universal screening in environments in which these adolescents are most likely to encounter mental- or physical-health services, such as schools and health-care settings.

When considered annually, the proportion of middle and high school age Mexican-origin girls reporting STBs in the past year was comparable with prior cross-sectional studies of U.S. Latina samples (Kann et al., 2014). Yet such single time-point assessments of suicidality in the past year may underestimate the true prevalence given that we observed that by the time they were in 12th grade, 38% of girls had reported STBs at least once since fifth grade and 20% reported STBs across multiple years. These figures reinforce concerns that adolescent suicidality has reached epidemic levels in the United States (Hoffmann et al., 2020), especially for Latinas (Price & Khubchandani, 2017; Zayas et al., 2005).

In addition, the prevalence of STBs in this sample of Mexican-origin female adolescents, mirroring that of U.S. Latina STB rates more broadly, was higher than that observed for similar-age females in Mexico (Borges et al., 2008). Combined with our finding that more U.S.-born Mexican-origin youths than first-generation youths reported STBs (Peña et al., 2008; SAMHSA, 2003), there is strong evidence that the U.S. national context is contributing to this public-health problem for Mexican-origin adolescents. Our data were collected from 2006 through 2014, which was a period of increasing deportations for Latino communities (Gonzalez-Barrera & Krogstad, 2016), yet it preceded the rapid escalation in antiimmigrant political rhetoric, detainment, and deportation that began in 2016 and has been linked to STBs in Latino youths (Roche et al., 2020). Although we did not assess youths’ perceptions of the national context for Mexican-origin families, these factors may have played a role in their mental health. Because the direct experience of discriminatory actions from peers and teachers did not account for these elevated rates of STBs, further examination is needed to comprehensively understand the mechanisms behind the links between later-generation status and increased risk for STBs (but see Marks et al., 2014).

Corroborating findings from prior studies (Lau et al., 2002; Van Geel et al., 2014; Villarreal-Otálora et al., 2020), we found that adolescents who experienced more conflict with peers and family were more likely to report STBs at multiple ages both when either risk factor was examined independently and when the psychosocial factors were considered collectively. The quality of close relationships with family and friends conveys robust influences on Mexican-origin youths’ well-being (CDC, 2004; Fortuna et al., 2007; Kuhlberg et al., 2010; Piña-Watson et al., 2014; Zayas & Pilat, 2008). The transition into high school, ninth grade to 10th grade, was a particularly salient period for the effects of peer conflict, which has previously been noted as a vulnerable age for stressors to trigger suicidality (Kann et al., 2018; Rew et al., 2016). School-based prevention and intervention programs that target peer stressors might yield the greatest benefit by focusing on periods of academic transition, which can disrupt existing relationships and social-support systems and reduce social status relative to older youths in the new school setting (Goldstein et al., 2015; Magson et al., 2021). Furthermore, with less adult supervision, the transition to high school may make some youths more susceptible to peer victimization and thus place them at greater risk for STBs.

The Mexican-origin youths in our study reported low rates of ethnic discrimination at school, which may have been attributable to the fact that most attended majority-Latino schools. In addition, the measures of ethnic discrimination and peer conflict were positively correlated. Peer conflict and bullying directed at immigrant and ethnic minoritized youths often emphasize differences in racial and ethnic background, language, and immigration status (Maynard et al., 2016; Peskin et al., 2007). This ethnic- or race-focused bullying could also be categorized as discrimination or racism, making this type of peer conflict especially noxious. It will be important for future research with Mexican-origin youths to contrast school and community contexts in which they experience numeric minority or majority status and to measure perceptions of both discriminatory and nondiscriminatory peer conflict to disentangle the unique contributions of each risk factor to STBs.

It was the familial measures, though, that most strongly predicted STBs by Mexican-origin adolescents; family conflict exacerbated risk, and familism values mitigated it. In addition, at multiple points in adolescence, both lower familism and greater family conflict were associated with increased risk for suicidality 1 year later, an important extension from previous cross-sectional and retrospective research (Kuhlberg et al., 2010; Prinstein et al., 2000). Relatively few empirical studies have evaluated how familism is connected to more salubrious outcomes in adolescents (Schriber et al., 2017). Researchers have argued that Latino individuals with high levels of familism exhibit collectivistic values, showing an enduring responsibility for the well-being of their families beyond that of the self (Cauce & Domenech-Rodriguez, 2002). This greater focus on the needs of close others may keep Mexican-origin adolescents from engaging in STBs because of heightened awareness of the impact of their own actions on their family members.

Negative relationship processes, particularly in the family, were key indicators of exacerbating STBs. Arguing with caregivers and being critiqued by family members for not participating in traditional cultural events or showing pride in their Mexican heritage were events captured by our measure of family conflict. This combination of relationship and cultural stressors in the family context may leave Mexican-origin adolescents feeling isolated from their primary support systems, making them especially vulnerable for STBs. In line with the cultural value of familism, the Latino family structure and connections among family members are thought to facilitate and strengthen individual well-being (Alegría et al., 2002), but this protective buffer may be disrupted by high levels of family conflict. The consistent, sustained, and predictive associations of familism and family conflict with STBs suggest that focusing on family tensions and connections could be promising targets of prevention and intervention efforts to reduce adolescent STBs in the Mexican-origin community (Goldston et al., 2008) and promote cultural identification to foster connectedness and support well-being (Berkel et al., 2010). The stability with which greater familism predicted decreased STBs across adolescence highlights the protective role of family connectedness and cultural identification for Mexican-origin youths (Ayón et al., 2010; Cruz et al., 2019; Schriber et al., 2017), even in the face of conflict and adversity in other realms of the adolescent’s life. Knowing the extent to which family processes play critical roles in promoting or undermining Mexican-origin adolescents’ mental health, future prevention and intervention efforts should leverage these cultural values to foster greater family connectedness and support caregivers to navigate conflict with their adolescents.

It should be emphasized that although all the psychosocial factors demonstrated independent and/or unique contributions to greater STBs in youths, in this unselected community sample, most participants did not endorse high levels of family conflict, peer conflict, and ethnic discrimination. Despite evincing lower variability and modest levels of risk, the analyses still demonstrated that even moderate adversity across different realms of life, especially involving interpersonal relationships, can lead to worse mental-health outcomes for Mexican-origin youths. Presumably, rates of STBs could be even higher in Mexican-origin and other Latino youths who experience more severe psychosocial adversities.

## Limitations and Future Directions

By collecting repeated measurements of STBs and its psychosocial predictors across adolescence and using LGC-modeling techniques to assess change over time in suicidality, in this study, we provide vital insights for mental-health researchers and practitioners working with the rapidly growing but understudied Mexican-origin community. Yet, as with all research, our findings must be considered in the context of certain study limitations.

First, in recognizing the diversity of the broader Latino community (Noe-Bustamante, 2019) and the structural and contextual differences in life circumstances for Latino families across the United States (Landale et al., 2006), it is important to acknowledge that these data were collected from the Mexican-origin population in California and thus may not reflect the experience of other Latino adolescents or even Mexican-origin communities in other U.S. states. Second, all measures of STBs and psychosocial predictors were derived from youths’ self-report. It would be beneficial for future research to also include reports by either parents or teachers, who would offer distinct accounts of youths’ behavior or adverse experiences. Third, some of our psychosocial questionnaires measuring negative events and interpersonal adversity produced modest internal consistency. This may be attributed to the relatively low prevalence of reported ethnic discrimination and interpersonal conflict among the participants, but in addition, these were formative constructs for which high interitem correlations are not expected such that metrics of internal consistency, such as coefficient alpha, are not ideal (Bollen & Bauldry, 2011; Bollen & Lennox, 1991; Markus & Borsboom, 2013). Despite lower variability among these measures, significant associations with concurrent and prospective STBs were identified. Fourth, our study was conducted from 2006 to 2014, and findings should be recognized as pertaining to that sociohistorical period in the United States. Forthcoming research would benefit from assessing youths’ perceptions of the national context for Mexican-origin families over the recent years of increasingly vitriolic public discourse and governmental policies and practices that have adversely affected this community. Given that the political climate and attitudes toward immigrants in the United States, especially individuals from Mexico and Central America, has deteriorated since these data were collected (Lopez et al., 2018), it is likely such a hostile and pervasive environment will have enduring adverse influences. Factors such as national policies, structural inequities, and systemic racism are likely to contribute to STBs among Mexican-origin youths and youths from other marginalized populations (Nazroo, 2003; Roche et al., 2020), and these warrant further attention. Finally, we recognize that suicidality is a multifactorial phenomenon that includes several contributing factors across domains, such as physiology, temperament, and other areas of psychopathology. Although this study examined prominent psychosocial and cultural variables known to contribute to risk for STBs (i.e., interpersonal conflict, ethnic discrimination), future research should include models that include multiple levels of analysis (e.g., Gene × Environment moderation) to capture both internal mechanisms and external influences.

## Conclusion

Although decades of research have examined factors associated with adolescent STBs, many questions remain about how to successfully identify which youths are at greatest risk and, consequently, how to design efficacious prevention programs, especially for Mexican-origin and other Latino communities. By longitudinally assessing suicidal ideation, planning, and past attempts in a nonclinical Mexican-origin sample from early to late adolescence, our study demonstrated that normative experiences in youths (e.g., fights with friends, difficulty with caregivers), even at modest levels, may be associated with experiences of suicidality at different points in development. Conversely, our work also suggests that experiences of connectedness to family and having a sense of familial obligation may promote greater mental health in Mexican-origin youths and thus buffer against psychopathology. Finally, we identified demographic factors (increased age, female sex, and generation status) that signaled risk for STBs across adolescence, which could be targeted in prevention efforts such as screening strategies.

No single known predictor has been identified that leads a youth to experience suicidal ideation or make a suicide attempt, but the more research can illuminate various risk factors that make a youth vulnerable for suicidality, the closer developmental and clinical scientists come to understanding this complex phenomenon. In conclusion, as researchers and practitioners seek to develop efficacious interventions for adolescent STBs, attention must be paid to the cultural values and relationship contexts of Mexican-origin and other Latino youths. Efforts to resolve tensions in the family and to promote Mexican-origin adolescents’ connectedness to their families and culture across development may help stem the rising tide of adolescent suicide in the United States.

## Transparency

*Action Editor:* Steve S. Lee

*Editor:* Jennifer L. Tackett

*Author Contributions*

Conceptualization: L. Gonzalves, P. D. Hastings. Data curation: A. E. Guyer, P. D. Hastings, R. W. Robins. Formal analysis: E. Ferrer, L. Gonzalves. Funding acquisition: A. E. Guyer, P. D. Hastings, R. W. Robins. Investigation: L. Gonzalves. Methodology: A. E. Guyer, E. Ferrer, L. Gonzalves, R. W. Robins. Project administration: P. D. Hastings. Visualization: L. Gonzalves. Writing–original draft: L. Gonzalves, P. D. Hastings. Writing–review & editing: A. E. Guyer, E. Ferrer, L. Gonzalves, P. D. Hastings, R. W. Robins.
